# Association of hyperdivergent facial pattern and the inclination of the occlusal plane in orthodontic patients: A cross-sectional study

**DOI:** 10.4317/jced.62910

**Published:** 2025-07-01

**Authors:** Marco Sánchez-Tito, Jorge Melgar-Gutiérrez, Ailín Cabrera-Matta

**Affiliations:** 1 Faculty of Public Health, Universidad Peruana Cayetano Heredia, Lima, Peru; 2 Faculty of Stomatology, Universidad Peruana Cayetano Heredia, Lima, Peru; 3 Department of Stomatology for Children and Adolescents, Universidad Peruana Cayetano Heredia, Lima, Peru

## Abstract

**Background:**

To evaluate the association between the hyperdivergent facial pattern and occlusal plane inclination in patients undergoing orthodontic treatment.

**Material and Methods:**

A retrospective cross-sectional study was conducted using records from patients who attended an orthodontic consultation at a specialized clinic in Tacna, Peru, between July 1, 2022, and August 31, 2023. Occlusal plane inclination was measured on lateral cephalometric radiographs following Andrews’ guidelines. Cephalometric analysis was performed using the OneCeph application. The vertical facial pattern was determined by the Frankfurt-Mandibular Plane Angle (FMA). A multiple linear regression analysis with robust variance was used to assess associations, considering *p*-values < 0.05 as statistically significant.

**Results:**

Occlusal plane inclination was significantly associated with vertical facial pattern (*p*< 0.001), with hyperdivergent individuals showing the greatest inclination. Additional significant correlations were observed with facial angle, convexity angle (*p*< 0.001), lower anterior facial height (*p* = 0.012), overjet (*p* = 0.042), and skeletal relationship (*p* = 0.013). Multiple regression confirmed greater occlusal plane inclination in hyperdivergent individuals compared to normodivergent (β = 2.15°, *p*< 0.001) and hypodivergent (β = 2.75°, *p*< 0.001) patterns.

**Conclusions:**

The hyperdivergent facial pattern was significantly associated with increased occlusal plane inclination, underscoring the importance of considering vertical growth patterns during orthodontic diagnosis and treatment planning.

** Key words:**Vertical facial pattern, Occlusal plane, Hyperdivergent.

## Introduction

Alterations in growth patterns are quite common in the general population. It is estimated that 45% of malocclusions are associated with those that have vertical involvement (1). The hyperdivergent vertical growth pattern requires complex orthodontic management, making its treatment important for both aesthetic and functional reasons. This growth pattern is typically established early in life, and its causes are linked to environmental factors, such as airway compromise and weak masticatory muscles (2). It leads to a posteroinferior rotation of the mandible, which compensates for changes in the dentoalveolar process (3).

Mohammadi *et al*. found that the normodivergent vertical facial pattern was the most common among a sample of Iranian patients, observed in 47.2% of cases, followed by the hyperdivergent pattern at 41.4% (4). In India, a study reported a prevalence of the hypodivergent pattern at 54.6%, with the hyperdivergent pattern following at 36.5% (5). Recently, research on Peruvian orthodontic patients estimated the prevalence of the hyperdivergent pattern to be 36.6%, while the normodivergent pattern was found in 33.5% of patients (6).

The high prevalence of malocclusions poses a public health concern, as they can lead to both functional and aesthetic issues that may impact individuals’ psychosocial development and overall quality of life (7). Research indicates that a hyperdivergent profile is considered less attractive compared to other vertical patterns, which can negatively affect patients’ social development (8).

Individuals with hyperdivergent facial characteristics typically exhibit increased facial height, a short mandibular ramus, a high gonial angle, and vertical enlargement of the maxilla (9,10). Moon *et al*. demonstrated that hyperdivergent patterns emerge early in life and become more pronounced with age, with the anterior facial height dimension being the most affected (11).

There is evidence suggesting that hyperdivergence influences the inclination of the occlusal plane (12). Kim *et al*. demonstrated that the rotation of mandibular growth is linked to the rotation of the occlusal plane, which occurs due to a more significant increase in vertical height at the molar level (13). Additionally, Tanaka *et al*. proposed that the position of the occlusal plane during facial growth is determined by the vertical growth of the maxillary teeth. They noted that the inclination of the occlusal plane is primarily influenced by the growth of the dentoalveolar bone, which compensates for mandibular rotation (12). Thus, vertical growth at the level of the condyle and the posterior segments of the maxilla are determining factors for the rotation of the mandible and can potentially generate changes in the inclination of the occlusal plane (3,14,15). However, other studies suggest an opposite relationship, where the occlusal plane’s control would impact the mandibular growth and rotation tendency, promoting expressions of modification of vertical and horizontal growth, mainly during growth periods (16-18).

The inconsistent findings regarding the relationship between vertical growth patterns and the inclination of the occlusal plane may stem from the methods used to evaluate the occlusal plane’s inclination (13,18). Relying on intracranial landmarks introduces inherent variability and is subject to biological variation, which can lead to errors in orthodontic treatment planning (19). Andrews and Andrews proposed using reference points on the forehead in relation to true vertical alignment to assess soft tissue, as well as skeletal and dental characteristics, including the occlusal plane (20-22). This approach is intended to reduce measurement errors.

Therefore, this investigation aimed to determine whether the hyperdivergent facial pattern is associated with the inclination of the occlusal plane in patients requiring orthodontic treatment.

## Material and Methods

- Study design, ethical considerations and statistical power

A retrospective cross-sectional study was conducted and received approval from the Institutional Committee of Ethics in Research at the Universidad Peruana Cayetano Heredia, with registration number CIEI-5-2-25. The research used a secondary database, so the calculation of statistical power was performed with the statistical program Stata V.18 (Stata Corp., College Station, TX, USA), considering the number of participants, a power = 0.8, the number of variables included in the model (n = 6), a minimum detectable R2 value of 0.056 and an alpha = 0.05. The calculated statistical power was 79.96%.

- Study location and participants

The source population was considered to be all patients who attended an orthodontic consultation at a specialized clinic in the city of Tacna - Peru, between July 1, 2022, and August 31, 2023. The selection criteria were participants with complete permanent dentition, without a history of previous orthodontic treatment, requiring orthognathic surgery, temporomandibular disorders, and chronic periodontal disease. During the initial appointment, the clinic routinely requests each patient to undergo a lateral skull and panoramic X-ray. Additionally, every patient is asked to sign a consent form allowing the use of their records for research purposes. All data collection and records management, including the identification of anatomical landmarks and cephalometric tracings, are conducted by a single operator with over ten years of experience as an orthodontist.

- Measurement of cephalometric magnitudes

Cephalometric radiographs were taken using Orthophos SL 3D radiographic equipment (Dentsply Sirona, Charlotte, NC, USA). The equipment operated at a nominal current of 12 A and a nominal power of 2 kW, with a voltage of 90 kV and an exposure time of 14.18 seconds. The voxel size was set at 80 μm.

**Figure 1:**
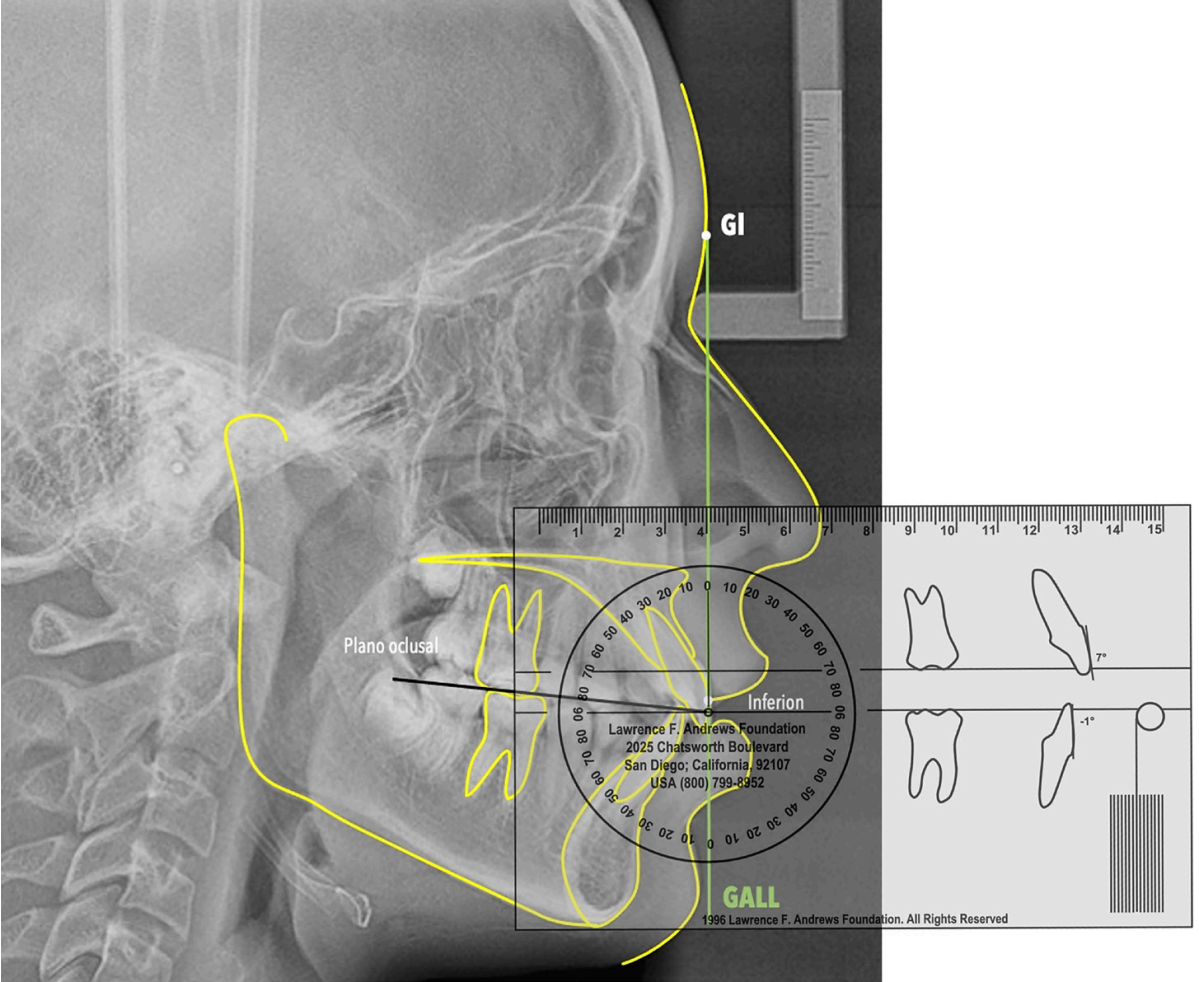
Measurement of the inclination of the occlusal plane in relation to GALL.

The occlusal plane is defined as a functional plane established by the midpoints of the molars and first premolars (14). Its inclination is measured in relation to a horizontal line that is perpendicular to the Goal Anterior Limit Line (GALL). According to Andrews, the normal values for the inclination of the occlusal plane range from +2° to +10° (22). To identify this inclination, a vertical line was drawn from the soft tissue glabella point onto acetate paper. This transfer process begins by measuring the distance from the central point of the clinical crown of the incisor (referred to as the FA point) relative to the glabellar vertical line using the frontal plane indicator (FPI) (23). This measured distance in millimeters is then marked on acetate paper as the location of the lower point. A line is drawn connecting the glabellar point (Gl) and the lower point; this line represents the GALL. Next, the midpoints of the molars and premolars are identified on the acetate paper, and a line is drawn to indicate the functional occlusal plane. This line is extended until it intersects the GALL. Finally, using the Andrews template, the angle formed between the occlusal plane and the horizontal line of the ruler, which is positioned perpendicular to the GALL, is measured (Fig. 1).

The cephalometric measurements were evaluated using radiographic images in JPG format through the free application OneCeph® (version beta 1.1, NXS, Hyderabad, India) (24). After calibrating the images, the anatomical reference points were identified, allowing for the calculation of angular and linear measurements generated by the application. The vertical facial pattern is defined as a characteristic that indicates the vertical growth direction of the face (25). To measure this, we followed Tweed’s recommendations, utilizing the FMA angle (Frankfurt-Mandibular Plane Angle) (26), which is the angle formed between the mandibular plane and the Frankfurt horizontal plane. Values less than 21° indicate a hypodivergent vertical pattern, values between 21° and 28° correspond to a normodivergent vertical pattern, and values greater than 28° are associated with a hyperdivergent vertical pattern.

- Theoretical model

**Figure 2:**
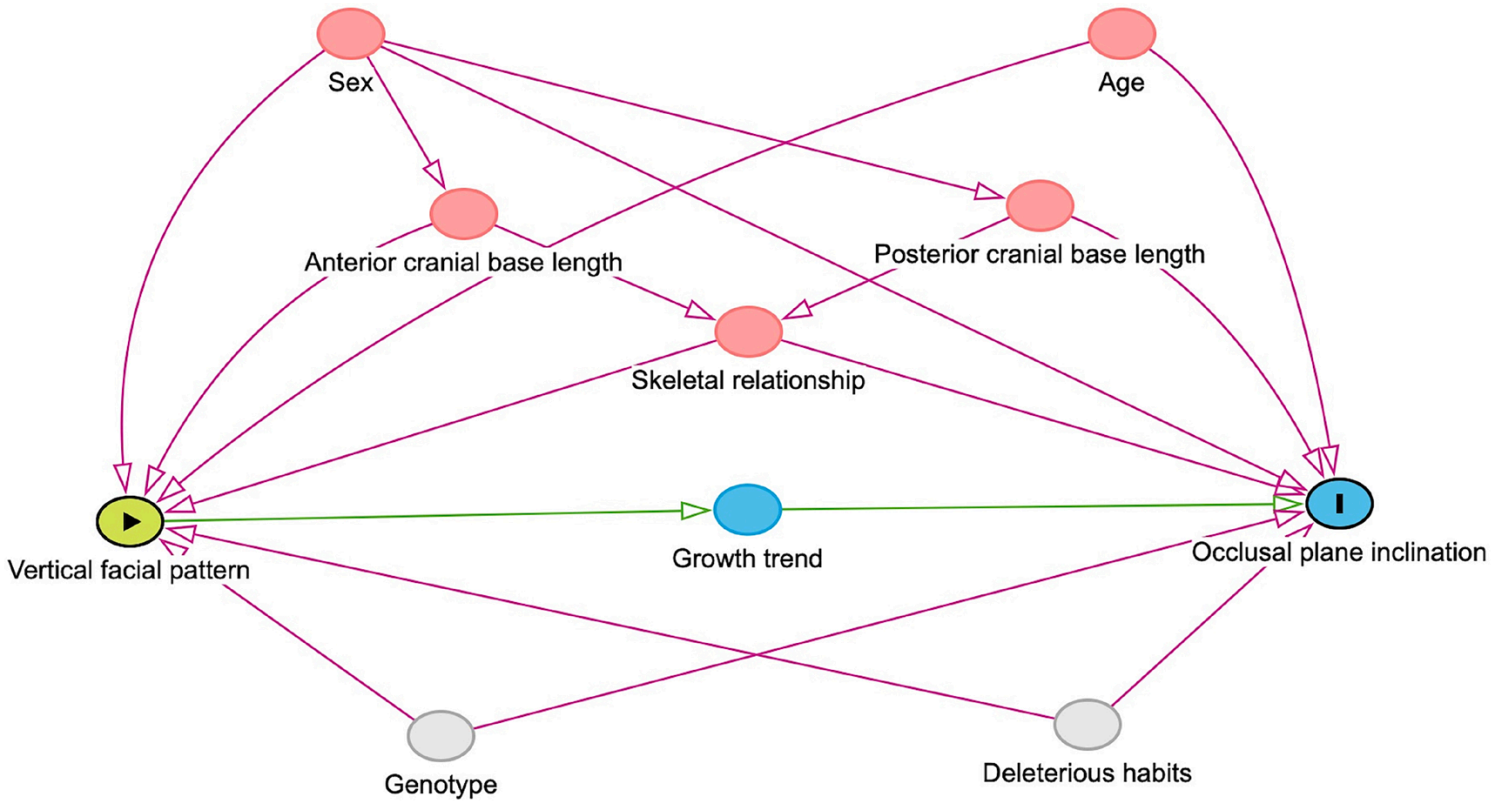
Directed acyclic graphs (DAG) showing the associations between exposure (vertical facial pattern) and outcome (occlusal plane inclination), confounders and mediator.

The main exposure factor being studied was the vertical facial pattern, which was analyzed as a categorical variable. The outcome of interest was the inclination of the occlusal plane, analyzed as a continuous variable. A directed acyclic graph (DAG) illustrating the association between the vertical facial pattern and the inclination of the occlusal plane was created using DAGitty v3.0 (http://dagitty.net). To avoid over-adjustment, we included only five confounding variables based on the literature: sex, age, skeletal relationship, anterior skull base length, and posterior skull base length (see Fig. 2). Other cephalometric measurements were analyzed as covariates during descriptive and exploratory bivariate analyses.

- Data analysis

Data were analyzed using Stata version 18 software (Stata Corp., College Station, TX, USA). Descriptive analysis of categorical variables included both absolute and relative frequencies. For numerical variables, we considered measures such as mean, median, standard deviation, and interquartile range. The normal distribution of numerical data was assessed using indicators like skewness and kurtosis, along with graphical evaluations using frequency histograms and qqplots. Depending on the nature of the variables, we employed the Student’s t-test, Spearman’s rank correlation, and one-way ANOVA for bivariate analysis. For multivariate analysis, a multiple linear regression with robust variance was conducted after confirming compliance with the necessary assumptions for this model. We performed both crude and adjusted model for confounders. The values of the β coefficients were analyzed, considering *p-value*s less than 0.05 as statistically significant, and 95% confidence intervals (95% CI) were reported.

## Results

**Figure 3:**
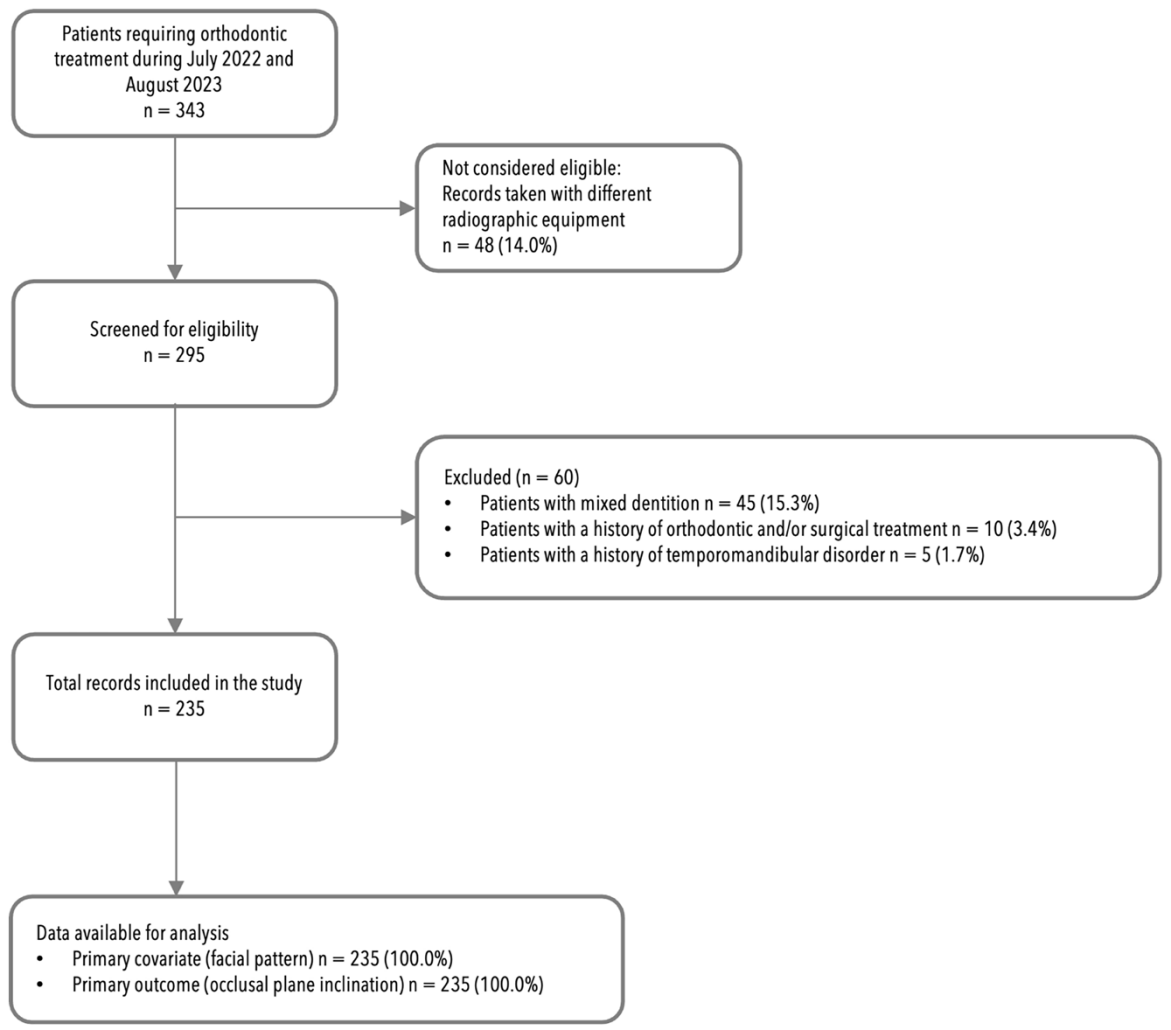
Flowchart of participant record selection.

A total of 343 eligible individuals were initially identified for the study. However, 14% of these individuals were excluded because their cephalometric radiographs were taken using different radiographic equipment. Additionally, 60 individuals did not meet the selection criteria for the study. The primary reasons for non-inclusion included the presence of mixed dentition in 15.3% of cases, a history of previous orthodontic and/or surgical treatment in 3.4%, and a history of temporomandibular disorder in 1.7%. Ultimately, 235 records, accounting for 79.6%, were retained for the study (Fig. 3).

This study involved 235 participants, comprising 60.0% women and 40.0% men, aged between 15 and 24 years, with a median age of 17 years (IQR 14-23).  Table 1 presents the distribution of the vertical facial patterns along with other cephalometric measurements of the participants. The most common vertical pattern identified was normodivergent, accounting for 49.4% of the participants, followed by hyperdivergent at 32.8%. Additionally, the mean inclination of the occlusal plane was measured at 9.2° ± 2.1°.

 Table 2 illustrates the relationships between various covariates and the inclination of the occlusal plane. A statistically significant association was found between occlusal plane inclination and vertical facial patterns (*p* < 0.001). Specifically, individuals with a hyperdivergent vertical facial pattern exhibited a greater occlusal plane inclination (10.7° ± 2.5°) compared to those with normodivergent (8.5° ± 1.3°) and hypodivergent (7.9° ± 1.4°) patterns. Additionally, significant correlations were identified between occlusal plane inclination and several cephalometric measurements: facial angle (*p* < 0.001), convexity angle (*p* < 0.001), antero-inferior facial height (*p* = 0.012), overjet (*p* = 0.042), and skeletal relationship (*p* = 0.013).

 Table 3 presents the β coefficients calculated using the simple and multiple linear regression models. Simple linear regression analysis showed that in participants the mean occlusal plane inclination in those with a hyperdivergent vertical pattern was significantly greater than in those with a normodivergent vertical pattern (β [95% CI] = 2.17° [1.66°, 2.69°]; *p* < 0.001). After adjustment for confounders, this difference remained significant (β [95% CI] = 2.15° [1.59°, 2.70°]; *p* < 0.001).

Additionally, a linear combination of parameters was performed to compare the hyperdivergent and hypodivergent vertical patterns; the results showed that the average inclination of the occlusal plane in the hyperdivergent was significantly greater than in the hypodivergent participants (β [95% CI] = 2.75° [2.03°, 3.48°]; *p* < 0.001) ( Table 4).

## Discussion

This study assessed whether individuals with a hyperdivergent vertical facial pattern have a greater inclination of the occlusal plane compared to those with normodivergent and hypodivergent patterns. The findings indicated that the hyperdivergent vertical pattern was significantly associated with a greater inclination of the occlusal plane than the other vertical patterns. Consequently, this investigation suggests that evaluating the relationship between vertical facial patterns and the occlusal plane is essential in orthodontic diagnosis. This approach could facilitate the development of personalized treatment strategies, including adjustments in vertical, transverse, and sagittal dimensions, ultimately leading to more predictable functional and aesthetic outcomes.

The hyperdivergent vertical growth pattern is established early in development (2), and it has been suggested that this pattern’s effect on the posterior-inferior rotation of the mandible influences the inclination of the occlusal plane (3,14,15). This inclination may play a role in orthodontic treatment planning decisions. A study conducted by Farhat *et al*. found that the inclination of the occlusal plane significantly differed between patients with a hyperdivergent vertical growth pattern and those with hypodivergent or normodivergent patterns. Furthermore, the upper anterior facial height seems to serve as a compensatory factor for the occlusal plane’s inclination in individuals with a hyperdivergent pattern (27).

An increase in antero-inferior facial height leads to a downward and backward rotation of the mandible, which results in an increased angle of the mandibular plane. These changes may cause a vertical adaptation in the dentoalveolar processes as a compensatory mechanism, thereby increasing the inclination of the occlusal plane (12-14).

In this study, it was found that 32.8% of the participants exhibited a hyperdivergent vertical facial pattern. A study conducted in India found that 36.5% of the sample presented this hyperdivergent vertical pattern, as assessed by the SN-GoGn angle (Sella-Nasion-Gnathion) (5). In Peru, it was estimated that the prevalence of the hyperdivergent vertical pattern among patients requiring orthodontic treatment, evaluated using the Ricketts Vertical Index, was 36.6% (6).

It’s important to note that, although the results of the studies were similar, they employed different methods for identifying the vertical growth pattern. As such, there is currently no consensus on a single method to determine the characteristics of this growth pattern. In this study, we utilized the FMA angle, which is the angle formed between the mandibular plane and the Frankfurt horizontal plane, as proposed by Tweed (26). This method is considered straightforward since it relies on only four anatomical points. This simplicity helps reduce errors that can arise from identifying anatomical landmarks in lateral skull radiographs, which may be affected by image superposition, as noted in other studies (19,28).

Research literature has examined the influence of sexual dimorphism on cephalometric measurements, focusing on the vertical patterns and morphological features of the mandible (15). Mangla *et al*. discovered that the height and width of the mandibular ramus were significantly lower in hyperdivergent individuals compared to those who are hypodivergent. Additionally, sexual dimorphism was more pronounced in men, who exhibited higher mandibular ramus than women (10). On the other hand, Jacob and Buschang noted that between the ages of 10 and 15, the increase in the palatal plane angle, as well as the ratio of anterior to posterior facial height and upper to lower height, was more pronounced in boys than in girls (29). In our study, bivariate analysis revealed that women had a significantly greater inclination of the occlusal plane compared to men; however, the impact of this variable was not significant in the adjusted regression model.

It has been suggested that the inclination of the occlusal plane is linked to the skeletal relationship. Specifically, individuals with a Class II skeletal relationship tend to have a more inclined occlusal plane, whereas those with Class III relationships exhibit a more horizontal occlusal plane (12,30). In a study by Li *et al*., it was found that the inclination of both the functional occlusal plane and the bisected occlusal plane was significantly steeper in patients with Class II skeletal relationships compared to those with Class I and Class III relationships, both before and after orthodontic treatment (*p*<0.001). Furthermore, both forms of occlusal plane inclination measurement were highly correlated before (r = 0.872) and after (r = 0.920) orthodontic treatment in this group of patients (31). This trend was also observed in our study, where individuals with a Class II skeletal relationship presented a greater inclination of the occlusal plane when compared to those with a Class I and Class III skeletal relationship. However, the differences detected were not significant when this variable was included in the adjusted model.

This retrospective study utilized data from cephalometric records of orthodontic patients, highlighting the potential bias arising from various unmeasured confounders. As a result, we were unable to evaluate the influence of other variables that may be associated with the inclination of the occlusal plane. These include deleterious habits during growth and development, such as mouth breathing, tongue interposition at rest, and sucking habits. Such factors can significantly impact the configuration of skeletal and dental relationships if not addressed early on. It is recognized that one of the factors linked to the development of Class II skeletal relationships is the presence of uncontrolled habits (32). This may explain why the association between the occlusal plane and the skeletal relationship was not significant in the adjusted model. The lack of control for this confounding variable likely affected the relationship between the other measured variables.

The cross-sectional nature of this study prevents the establishment of a causal relationship between the facial vertical pattern and the inclination of the occlusal plane. Additionally, the findings have limitations when it comes to generalizing to the broader population, as the study only included individuals with significant malocclusions who required orthodontic treatment. Therefore, the results do not necessarily represent the entire population.

## Conclusions

Individuals with a hyperdivergent vertical facial pattern exhibited a greater inclination of the occlusal plane compared to those with normodivergent and hypodivergent patterns. These findings indicate that assessing the relationship between vertical facial patterns and the occlusal plane is important for orthodontic diagnosis.

## Figures and Tables

**Table 1:** Characteristics and cephalometric magnitudes of the participants (n=235).

Characteristics/Cephalometric magnitudes	n (%)
Sex	
Female	141 (60.0)
Male	94 (40.0)
Age (years)*	17 [14-23]
Facial angle (°)	88.1 ± 4.0
Angle of convexity (°)	7.7 ± 7.5
Anterior cranial base length (°)*	61 [59-64]
Posterior cranial base length (°)	32.2 ± 3.7
Anterior lower facial height (mm)	66.6 ± 5.9
Posterior facial height (mm)*	74.4 [71-79.4]
Overjet (mm)*	2 [1.5-3]
Overbite (mm)*	1.5 [1-2]
Skeletal relationship	
Class I	91 (38.7)
Class II	116 (49.4)
Class III	28 (11.9)
Vertical facial pattern	
Hypodivergent	42 (17.9)
Normodivergent	116 (49.4)
Hyperdivergent	77 (32.8)
Occlusal plane inclination (°)	9.2 ± 2.1

* Median [percentile 25-75]. † Mean ± standard deviation.

**Table 2:** Characteristics associated with the inclination of the occlusal plane in the bivariate analysis (n=235).

Characteristics/Cephalometric magnitudes	Occlusal plane inclination	p
	(n=235)	
	Mean ± SD	
Sex*		0.034
Female	9.42 ± 2.19	
Male	8.82 ± 1.92	
Age (years)	-0.009	0.883
Facial angle (°)	-0.2	<0.001
Angle of convexity (°)	0.2	<0.001
Anterior cranial base length (mm)	-0.02	0.755
Posterior cranial base length (mm)	-0.11	0.094
Anterior lower facial height (mm)	0.2	0.012
Posterior facial height (mm)	-0.11	0.100
Overjet (mm)	0.13	0.042
Overbite (mm)	-0.07	0.256
Skeletal relationship		0.013
Clase I	8.9 ± 1.8	
Clase II	9.6 ± 2.3	
Clase III	8.3 ± 1.6	
Vertical facial pattern		<0.001
Hypodivergent	7.9 ± 1.4	
Normodivergent	8.5 ± 1.3	
Hyperdivergent	10.7 ± 2.5	

SD: Standard deviation * Student´s t-test † Spearman correlation coefficient ‡ One-way ANOVA

**Table 3:** Association between occlusal plane inclination (°) and vertical facial pattern.

Characteristics/Cephalometric magnitudes	Unadjusted			Adjusted*		
		IC 95%	p		IC 95%	p
Vertical facial pattern						
Normodivergent	Ref.			Ref.		
Hypodivergent	-0.62	-1.25, 0.17	0.056	-0.59	-1.26, -0.07	0.077
Hyperdivergent	2.17	1.66, 2.69	<0.001	2.15	1.59, 2.70	<0.001
Sex						
Female	Ref.			Ref.		
Male	-0.59	-1.14, -0.04	0.034	-0.37	-0.91, 0.15	0.162
Age	0.01	-0.04, -0.05	0.757	-0.01	-0.04, 0.03	0.772
Anterior cranial base length	-0.03	-0.09, -0,4	0.417	0.06	-0.01, 0.13	0.088
Posterior cranial base length	-0.06	-0.14, 0.01	0.083	-0.04	-0.12, 0.03	0.245
Skeletal relationship						
Class I	Ref.			Ref.		
Class II	0.65	0.08, 1.22	0.025	0.12	-0.39, 0.63	0.638
Class III	-0.57	-1.45, 0.31	0.201	-0.11	-0.89, 0.66	0.775

*Adjusted for sex, age, anterior cranial base length, posterior cranial base length, and skeletal relationship. β: β coefficient. 95% CI: 95% confidence interval

**Table 4:** Association between the inclination of the occlusal plane and the hypodivergent and hyperdivergent vertical pattern.

Characteristics/Cephalometric magnitudes	Unadjusted			Adjusted*		
		IC 95%	p		IC 95%	p
Vertical facial pattern						
Hypodivergent	Ref.			Ref.		
Hyperdivergent	2.79	2.11, 3.47	<0.001	2.75	2.03, 3.48	<0.001

*Adjusted for sex, age, anterior cranial base length, posterior cranial base length, and skeletal relationship. β: β coefficient. 95% CI: 95% confidence interval

## Data Availability

The datasets used and/or analyzed during the current study are available from the corresponding author.
